# Master Curve Establishment and Complex Modulus Evaluation of SBS-Modified Asphalt Mixture Reinforced with Basalt Fiber Based on Generalized Sigmoidal Model

**DOI:** 10.3390/polym12071586

**Published:** 2020-07-17

**Authors:** Guojin Tan, Wensheng Wang, Yongchun Cheng, Yong Wang, Zhiqing Zhu

**Affiliations:** 1 College of Transportation, Jilin University, Changchun 130025, China; tgj@jlu.edu.cn (G.T.); chengyc@jlu.edu.cn (Y.C.); zhuzq19@mails.jlu.edu.cn (Z.Z.); 2 Jilin Province Highway Administration Bureau, Changchun 130021, China; wangyjlgl@gmail.com

**Keywords:** asphalt mixture, basalt fiber, complex modulus, master curve

## Abstract

Basalt fiber has been proved to be a good modified material for asphalt mixture. The performance of basalt fiber modified asphalt mixture has been widely investigated by extensive researches. However, most studies focused on ordinary static load tests, and less attention was paid to the dynamic mechanical response of asphalt mixture incorporating with basalt fiber. This paper aims to establish the master curve of complex modulus of asphalt mixture incorporating of styrene-butadiene-styrene (SBS) polymer and basalt fiber using the generalized Sigmoidal model. Both loading frequency and temperature were investigated for dynamic mechanical response of asphalt mixture with basalt fiber. In addition, based on the time-temperature superposition principle, the master curves of complex modulus were constructed to reflect the dynamic mechanical response at an extended reduced frequency range at an arbitrary temperature. Results indicated that the generalized Sigmoidal model in this paper could better reflect the dynamic mechanical response accurately with correlation coefficients above 0.97, which is utilized to predict the dynamic mechanical performances accurately. Simultaneously, the modulus values exhibit an increasing trend with loading frequency and decrease versus temperature. However, the phase angle values showed different trends with frequency and temperature.

## 1. Introduction

With the rapid increase of traffic, ordinary asphalt pavements often fail to meet the performance requirements, resulting in a few destructions including low and medium-temperature cracking, high-temperature rutting and freeze-thaw (F-T) destruction, and so on [1,2,3,4]. To improve mechanical performances, various additives such as rubbers, polymers, fibers and other additive materials have been adopted to incorporate with asphalt [5,6,7,8]. Studies demonstrated that polymers including styrene-butadiene-styrene (SBS), styrene-butadiene rubber (SBR), etc. have been proved to improve the high-temperature rutting, moisture damage and so on [9]. In addition, adding fibers to asphalt mixtures usually increases the mechanical performances, such as cracking resistance [10] To improve the compressive capabilities of asphalt materials effectively, researchers have made lots of efforts and tried many novel additives.

Most published studies have investigated SBS modified asphalt systematically [11,12,13]. Today, the SBS polymer modifiers are commonly applied in modified bitumen, and the demand for SBS modifiers is increasing with the development of highway construction. Imaninasab [14] investigated and evaluated the influences of the modification process of two kinds of polymers, i.e., SBS and ethylene-copolymer-bitumen (ECB), on anti-rutting performance at a high temperature of stone mastic asphalt (SMA). It was indicated that the elastic modulus of asphalt mixtures would reduce with SBS content increasing. Wang et al. [15] explored the experimental methods of polymer modified asphalt (PMA), including SBS with different proportions systematically, so as to ensure quality and requirement of construction engineering in asphalt pavement. Hajikarimi et al. [16] investigated the rheological and mechanical performances of SBS modified bitumen, as well as its binder, with three proportions by using rheometer equipment for the purpose of analyzing the storage and loss modulus and viscoelastic behavior. Furthermore, they used the obtained dynamic test results in the finite element software ABAQUS, to simulate the viscoelastic behavior of neat and SBS modified asphalt, respectively [17].

In recent years, basalt fiber has gained more and more attention for improving asphalt mixture, and many scholars have been devoting to study the influences of basalt fiber on the performance improvement of asphalt materials [18,19]. As a novel environmentally friendly mineral fiber, basalt fiber has several excellent advantages including better strength, high temperature, as well as acid and alkali resistance [20]. Thus, it has been proved that the incorporation of basalt fiber into bitumen has a better reinforced and bridging effects. Sun et al. [21] explored an enhancement impact of basalt fiber on toughness of asphalt materials. Test results indicated that the asphalt mixtures with a fiber content of 0.4% by weight would be better. Qin et al. [22] investigated the impacts of basalt fibers with various sizes and contents on asphalt mastics, and compared them with other common fibers, such as lignin fiber and polyester fiber. They drew a conclusion that basalt fiber has the best comprehensive performances and basalt fiber with 6 mm was suggested, due to its larger contact area with asphalt. Li et al. [23] conducted three-point bending tests at three low temperatures on the asphalt concretes AC-13 and AC-20 with various basalt fiber contents, and proposed a distinction method of fracture type based on the bending coefficient.

Most researchers tried to employ the incorporation of polymers and fibers into asphalt mixture to improve its comprehensive performance. Gu et al. [24] pointed out that SBS modified asphalt reinforced with basalt fiber has a higher rutting factor compared with original asphalt, based on dynamic shear rheological tests and repeated creep tests. It was reported that the high-temperature improvement effect of basalt fiber was significant compared with commonly used fibers. Tanzadeh et al. [25] investigated the open-graded friction course (OGFC) modified by polymer and basalt fiber by a drainage test and a common mechanical test. The test results revealed that OGFC with 0.2% basalt fiber and 4.5% SBS polymer had batter performances, and basalt fiber had a positive effect on reducing the draindown phenomenon. Miao et al. [26] examined four types of fibers and four types of asphalt including neat asphalt and polymers modified asphalt based on the interfacial properties. The results indicated that basalt fiber had the prime reinforcement and SBS modified asphalt was found to be well reinforced with fiber. Luo et al. [27] evaluated the enhancement impact of SBS and basalt fiber on anti-rutting and anti-cracking of modified asphalt mixture by the Hamburg wheel track test and the low temperature bending test, respectively. Kou et al. [28] selected basalt fiber to reinforce asphalt materials incorporating of SBS polymer, and they found that asphalt materials incorporating of SBS polymer and basalt fiber can make use of both advantages of additives [29,30,31]. Badeli et al. [32] explored the influences of F-T actions on fatigue cracking of asphalt mixtures considering seasonal ambient temperature variations. Cheng et al. [33] made an overall assessment of the mechanical performances of asphalt materials incorporating of basalt fiber and analyzed the improvement impact of F-T resistance based on volumetric and mechanical parameters. Furthermore, they analyzed logistic F-T damage models of asphalt mixtures and established a multi-variable grey model [34]. Cheng et al. [35] established a damage evolution of the mechanical performance of asphalt mixtures exposed to repeated F-T actions through reliability and damage theory, and predicted and analyzed its internal damage degradation. Tarefder et al. [36] analyzed the influences of F-T action on asphalt materials using several experimental methods on fatigue and rheometer. Test results showed that the fatigue life and creep stiffness of asphalt materials decreased due to the action of F-T cycles. Nevertheless, many efforts for asphalt materials incorporating of SBS polymer and basalt fiber have been made. Moreover, the dynamic mechanical responses of asphalt mixture need to be further illustrated.

Therefore, to explore the dynamic mechanical responses of asphalt materials incorporating of SBS polymer and basalt fiber, the complex modulus test was conducted at various temperatures and frequencies. Meanwhile, the influence of loading frequency and temperature on dynamic mechanical response of asphalt mixture was studied. In addition, based on time-temperature superposition principle, the master curves of complex modulus were established using the generalized Sigmoidal model, to reflect the dynamic mechanical response. The research results will provide some references for the further development and application of asphalt mixture with basalt fiber.

## 2. Formulations of Complex Modulus

### 2.1. Dynamic Mechanical Response of Viscoelastic Materials

**Figure 1:**
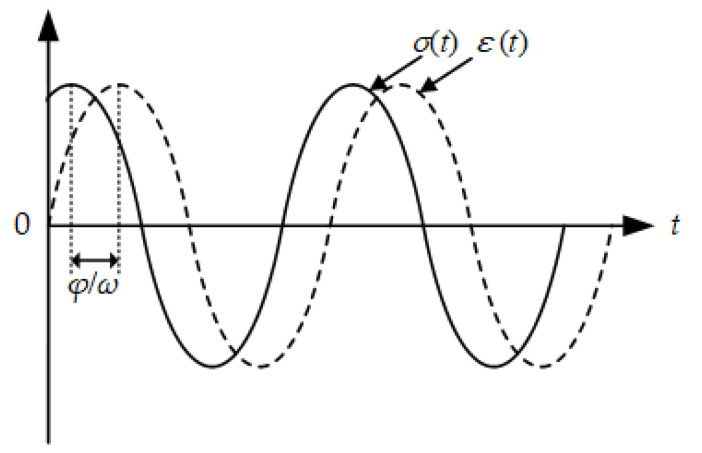
Curves of stress and strain in complex modulus test.

Dynamic mechanical analysis (DMA) is an increasingly important method for analyzing the mechanical behavior of viscoelastic materials, in which the complex modulus test is a dynamic test method commonly used in asphalt material testing. When asphalt materials are applied to a sinusoidal stress force (or sinusoidal strain load), its corresponding sinusoidal strain response (or sinusoidal stress response) is generally obtained, which is called a stress (or strain) controlled test. For a specimen under an applied cyclically varying sinusoidal load, its strain response would show a cyclically varying trend, but the strain response always lags the applied stress, which is the so-called stress-strain lag phenomenon, as plotted in Figure 1.

In a strain-controlled test, the applied strain-controlled load in a complex plane can be expressed as:

*ε*(*t*) = *ε*_0_ (cos*ωt* + *i*sin*ωt*) = *ε*_0_*e^iωt^*, (1)

where *ε*_0_ is the strain amplitude, *i* is an imaginary number of complex number, *ω* is angular frequency.

Then the stress response can be expressed as:

*σ*(*t*) = σ* *e^iωt^* = *σ*_0_*e^i^*^(*ωt* +^*^ϕ^*^)^, (2)

where σ* is a complex number of stress response amplitude, *σ*_0_ is an absolute value of complex number σ*, *ϕ* is a phase angle.

According to the viscoelastic differential constitutive model, the relationship between E*(*ω*) and *E*(*t*) could be obtain by combining Equations (1) and (2):

(3) $$E^{*}(\omega )=i\omega \bar{E}(i\omega ),$$

Equation (4) is expressed as a complex number in its frequency domain:

(4) $$E^{*}(\omega )=E^{\prime }(\omega )+iE^{\prime \prime }(\omega )=\left|E^{*}(\omega )\right|(\cos\varphi +i\sin\varphi )=\left|E^{*}(\omega )\right|e^{i\varphi },$$

where the real part $E^{\prime }(\omega )$ of complex modulus E*(*ω*) is the stored energy of viscoelastic materials under alternating stress, i.e., storage modulus, representing the elastic portion; the imaginary part $E^{\prime \prime }(\omega )$ is the energy dissipated as heat, representing the viscous portion. Its absolute value of E*(*ω*), $\left|E^{*}(\omega )\right|=\sqrt{{E^{\prime }}^{2}+{E^{\prime \prime }}^{2}}$, is dynamic modulus.

The tangent of the phase angle (tan*ϕ*) between stress and strain can be expressed as follows:

(5) $$\tan\varphi =\frac{E^{\prime \prime }(\omega )}{E^{\prime }(\omega )},$$

the tangent of the phase angle (tan*ϕ*) provides a measure of damping in the material.

### 2.2. Time-Temperature Superposition Principle

As a typical viscoelastic material, the viscoelastic properties of asphalt materials have an obvious dependence on time and temperature. In polymer physics, the time-temperature superposition principle is usually adopted to analyze the properties at unknown conditions, based on the properties at known conditions. Then, a master curve at a specified condition would be calculated and adopted to analyze the properties in a larger condition range, which would greatly reduce the test workload.

The translation distance of dynamic modulus curve at different temperatures to a reference temperature is called shift factor (*α_T_*), which is expressed as follows:

(6) $$\alpha_{T}=\frac{f}{f_{r}},$$

where *f* is a loading frequency at any temperature, *f_r_* is corresponding reduced frequency at a reference temperature.

At present, three commonly used time-temperature shift factor equations are selected to determine and establish the master curve, including Williams-Landel-Ferry (WLF) equation, Arrhenius equation and Log-linear equation.

1. Williams-Landel-Ferry (WLF) Equation (7) [37]:
  (7) $$\log\alpha_{T}=-\frac{C_{1}(T-T_{r})}{C_{2}+(T-T_{r})},$$
  where *C*_1_ and *C*_2_ are fitting parameters, *T* is test temperature and *T_r_* is reference temperature.
2. Arrhenius Equation (8) [38]:
  (8) $$\alpha_{T}=A\cdot e^{\frac{E}{R}(\frac{1}{T}-\frac{1}{T_{r}})},$$
  where *A* is material constant, *E* is the activation energy, *R* is gas constant, and usually selected as 8.314 J/(mol·K).
3. Log-linear equation (9) [39]:
  (9) $$\log\alpha_{T}=k(T-T_{r}),$$
  where *k* is the slope of fitting straight line for log*α_T_* versus *T*.

### 2.3. Construction of Master Curves Based on Generalized Sigmoidal Model

Based on the theory of time-temperature superposition principle, the viscoelastic characteristics of asphalt mixtures could be analyzed in a wider condition range. The establishment of a master curve shows the effect of frequency in a wider range of frequency. The determination of shift factor (*α_T_*) is the key to time-temperature superposition principle. In this paper, the WLF equation in Equation (7) is employed to calculate the shift factor (*α_T_*).

In general, the master curve of asphalt materials can be characterized by using the Sigmoidal model, i.e., the S-shaped growth model. However, the standard Sigmoidal model is only applicable to the case that data points are symmetrical with respect to the turning point of master curve. In addition, this standard Sigmoidal model ignores loss modulus and phase angle, resulting in Sigmoidal model to be inconsistent with actual test results. In this paper, the generalized Sigmoidal model shown in Equation (10) is utilized to establish master curves of dynamic modulus of asphalt mixtures [40].

(10) $$\mathrm{lg}\left|E^{*}\left(f_{r}\right)\right|=\delta +\frac{\alpha -\delta }{{\left(1+\lambda \cdot e^{\beta +\gamma \mathrm{lg}f_{r}}\right)}^{\frac{1}{\lambda }}},$$

where $\mathrm{lg}\left|E^{*}\left(f_{r}\right)\right|$ is dynamic modulus in logarithmic coordinates, *δ* is value of the lower asymptote of dynamic modulus |E*|, *α* is value of the upper asymptote of dynamic modulus |E*|, *λ*, *β* and *γ* are shape factors.

Based on linear viscoelastic theory, the two parts expressed in Equation (4) of complex modulus of asphalt materials usually need to satisfy the Kramers-Kronig (K-K) relationship [41]. According to Equation (10) and K-K relationship, the semi-log generalized Sigmoidal model can be derived for the master curve of phase angle, as presented in Equation (11).

(11) $$\varphi \left(f_{r}\right)=-\frac{\pi }{2}\cdot \frac{(\alpha -\delta )\gamma e^{\beta +\gamma \mathrm{lg}f_{r}}}{{\left(1+\lambda \cdot e^{\beta +\gamma \mathrm{lg}f_{r}}\right)}^{\left(1+\frac{1}{\lambda }\right)}},$$

where $\varphi \left(f_{r}\right)$ is phase angle in logarithmic coordinates, *δ* is value of the lower asymptote of phase angle, *α* is value of the upper asymptote of phase angle, *λ*, *β* and *γ* are shape factors.

Similarly, according to linear viscoelastic theory and K-K relationship, the generalized Sigmoidal model can be adopted to construction the master curves of storage modulus and loss modulus of asphalt material, which are shown in Equations (12) and (13).

(12) $$\mathrm{lg}\left|E^{\prime }\left(f_{r}\right)\right|=\delta +\frac{\alpha -\delta }{{\left(1+\lambda \cdot e^{\beta +\gamma \mathrm{lg}f_{r}}\right)}^{\frac{1}{\lambda }}},$$

(13) $$\begin{array}{ll} \mathrm{lg}\left|E^{\prime \prime }\left(f_{r}\right)\right| & =\mathrm{lg}\left(-\frac{\pi }{2}\cdot \left(\alpha -\delta \right)\cdot \gamma \right)+\left(\beta +\gamma \cdot \mathrm{lg}f_{r}\right)\mathrm{lg}e\\ & -\left(1+\frac{1}{\lambda }\right)\mathrm{lg}\left(1+\lambda \cdot e^{\beta +\gamma \mathrm{lg}f_{r}}\right)+\delta +\frac{\alpha -\delta }{{\left(1+\lambda \cdot e^{\beta +\gamma \mathrm{lg}f_{r}}\right)}^{\frac{1}{\lambda }}} \end{array}$$

## 3. Experimental Procedures

### 3.1. Raw Materials and Specimen Preparation

#### 3.1.1. Raw Materials

In this paper, the asphalt binder was SBS modified asphalt, which is a commonly used bitumen type in asphalt pavement. It was provided by Yingkou, China. The aggregates were crushed basalt graded aggregates. The mineral powder was ground by limestone. Eco-friendly basalt fiber was employed as fiber stabilizer. The physical parameters of these raw materials meet the requirements and they are listed in Table 1, Table 2, Table 3 and Table 4 [42].

#### 3.1.2. Specimen Preparation

**Figure 2:**
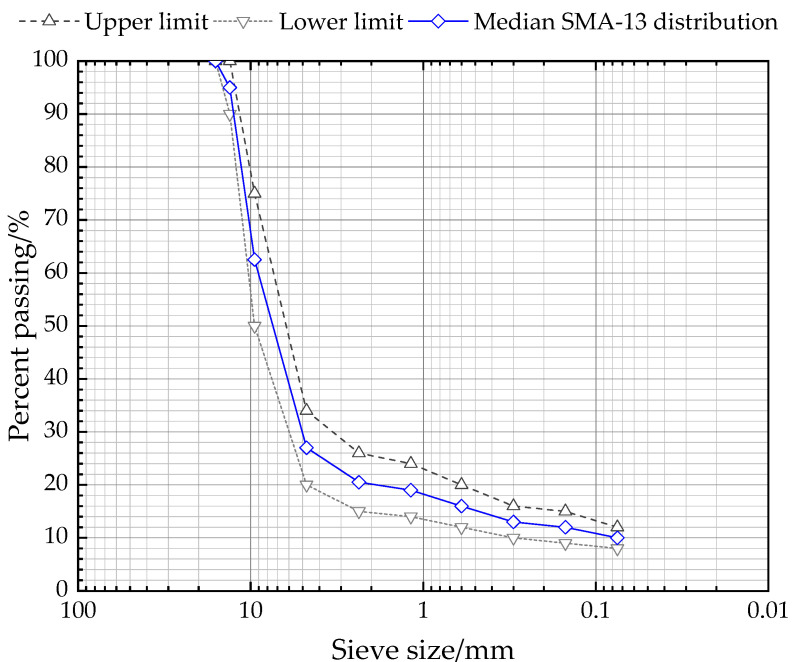
Gradation of stone mastic asphalt (SMA)-13.

Stone mastic asphalt (SMA) is a widely used asphalt mixture type composed of high-content coarse aggregate, mineral powder and asphalt, as well as low-content fine aggregate, which was initially formed in Germany in the 1960s and first applied in China in 1992 [8] Due to its better resistance to deformation and durability, SMA has been extensively applied for most pavement surfaces of highways in China. In this paper, the median gradation SMA-13 distribution illustrated in Figure 2 was chosen for manufacturing samples.

**Figure 3:**
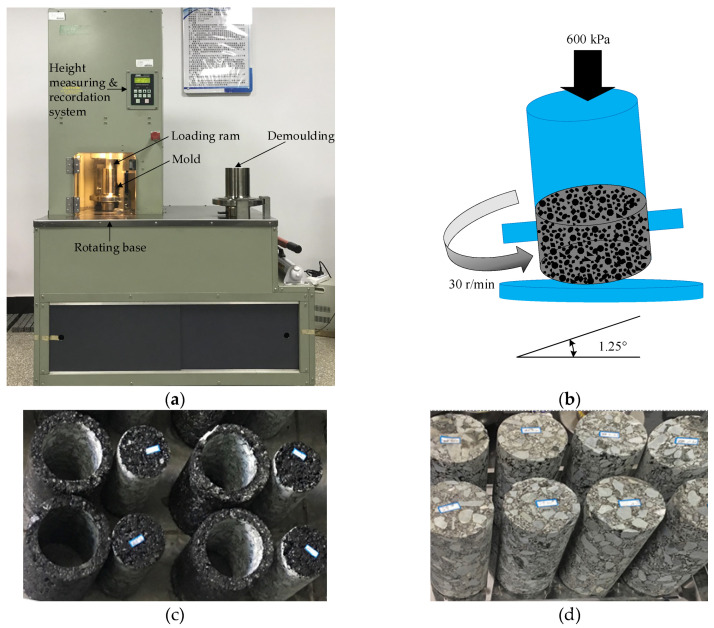
Diagram of Superpave gyratory compaction (SGC) in this paper: (**a**) Superpave gyratory compaction; (**b**) parameters; (**c**) SGC specimens and (**d**) core samples.

The sample preparation procedure followed the Chinese standard JTG E20-2011 [42]. There are several commonly used molding methods, including Marshall Compaction, Superpave gyratory compaction (SGC), roller compaction, static pressure molding and so on. As one of the important technical achievements of strategic highway research program (SHRP), SGC is a novel Superpave mixture design and molding method. Furthermore, it has been proved that the internal structures of SGC asphalt mixture specimens are consistent with core specimens from an actual road with a good correlation, which have less porosity variability. To better simulate the actual pavement construction, the SGC method (shown in Figure 3a) was selected to prepare asphalt mixtures with basalt fiber in this study. The detailed procedure of specimen preparation has been described in the previous studies [7,8]. The set SGC parameters are also illustrated in Figure 3b. Then, asphalt mixture specimens were first manufactured following the SGC method. After de-molding and cooling, asphalt mixture samples (diameter: 100 mm, height: 150 mm) could be prepared through using a core drilling machine and a cutting machine, as shown in Figure 3c,d.

### 3.2. Test Configuration and Protocol

**Figure 4:**
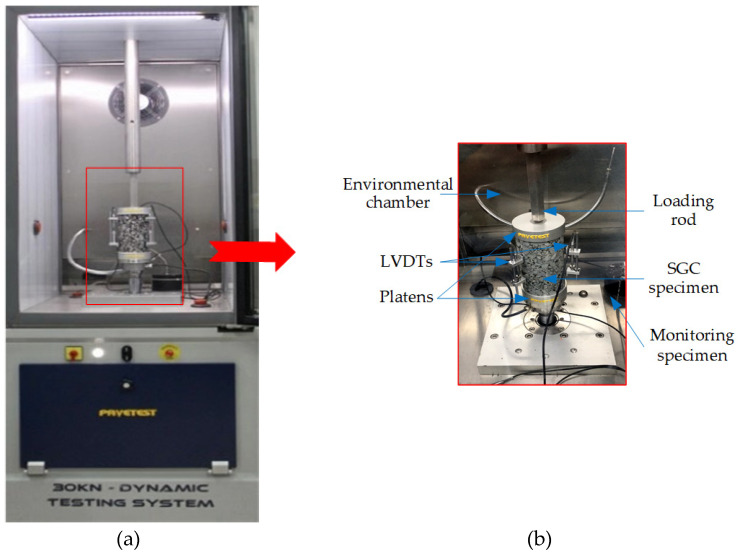
Complex modulus test used in this paper: (**a**) Dynamic testing system (DTS), (**b**) complex modulus test.

The complex modulus test is a common dynamic experimental method commonly used in asphalt mixture testing. In this paper, a dynamic testing system (DTS) with 30 kN servo-hydraulic and 100 mm range was employed to perform complex modulus tests according to the specification of AASHTO TP 79 [43]. The DTS with an environmental chamber has a loading range of ±16 kN, a frequency of up to 70 Hz, and controlled within −20 °C to 80 °C. Before the complex modulus test, equipped samples were kept in a testing chamber to equilibrate to the specified condition, as illustrated in Figure 4a, in which a monitoring sample was used as a reference. Three linear variable differential transformer (LVDT) brackets were placed and glued to asphalt mixture specimens at three locations 120 degrees apart, as illustrated in Figure 4b. During the complex modulus test, the test sample was applied to a haversine compressive force in a cyclic manner. The complex modulus tests were also carried out at specific conditions following the specification of AASHTO TP 79 [43]. The three vertical LVDTs could record the real-time axial strains of asphalt mixture specimens, which would be adjusted between 70 and 120 microstrains by the DTS automatically. After that, the corresponding mechanical test results could be obtained by the DTS software Testlab.

## 4. Results and Discussion

Based on the complex modulus test data for the 30 combinations of temperature and frequency, its normal value |E*| and phase angle *ϕ* could be derived by Equations (14) and (15).

(14) $$\left|E^{*}\right|=\frac{\sigma_{0}}{\varepsilon_{0}},$$

(15) $$\varphi =\frac{t_{i}}{t_{p}}\times 360,$$

where *σ*_0_ and *ε*_0_ are magnitude of axial stress and strain, respectively; *t_i_* is the average lag time between deformation peak and load peak; *t_i_* is the average loading period.

### 4.1. Determination of Dynamic Modulus of Asphalt Mixture Reinforced with Basalt Fiber

#### 4.1.1. Influence Analysis of Temperature and Frequency on Dynamic Modulus

**Figure 5:**
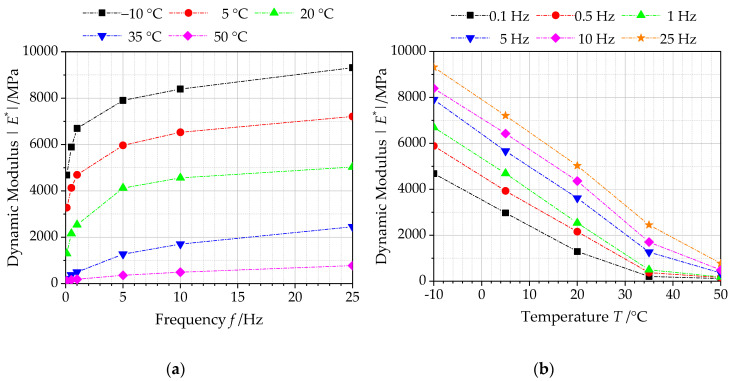
Dynamic modulus of asphalt mixtures with basalt fiber: (**a**) temperature, (**b**) frequency.

Figure 5 presents the dynamic modulus |E*| versus temperature and frequency for asphalt mixtures incorporating of SBS polymer and basalt fiber. As observed in Figure 5a, whatever the test temperature is, the dynamic modulus values of asphalt mixture samples exhibit an increasing trend with loading frequency. This is because as a viscoelastic material, asphalt mixture cannot be deformed immediately under dynamic load. Furthermore, it will need a period of rebound deformation during the unloading process. Under the same load, the dynamic deformation response would change with loading frequency, affecting the dynamic modulus. Considering the actual road conditions, it could be clearly observed that rut disease is more likely to occur in low speed areas, such as intersections, bus stops and parking lots. This could be explained by that asphalt pavement has a smaller dynamic modulus value under a lower loading frequency caused by low-speed cars.

It can be observed from Figure 5b that the dynamic modulus values of asphalt mixtures incorporating of SBS polymer and basalt fiber decrease with the increasing of test temperature. For different loading frequencies, the dynamic modulus of asphalt mixtures incorporating of SBS polymer and basalt fiber is the highest at the low temperature (−10 °C). This indicates that asphalt mixture will hardly deform under load and it is closer to a linear elastomer, while asphalt mixture is prone to cracking and failure under a long-term load. Simultaneously, as the experimental temperature increases, the elastic effect of asphalt materials incorporating of SBS polymer and basalt fiber gradually weakens, and its viscosity effect gradually enhances. Then asphalt pavement would be prone to a large permanent deformation under the heavy load. Thus, dynamic modulus of asphalt materials incorporating of SBS polymer and basalt fiber at a high temperature can be regarded as an evaluation indicator of high-temperature stability of asphalt pavement.

#### 4.1.2. Construction of Master Curves of Dynamic Modulus

**Figure 6:**
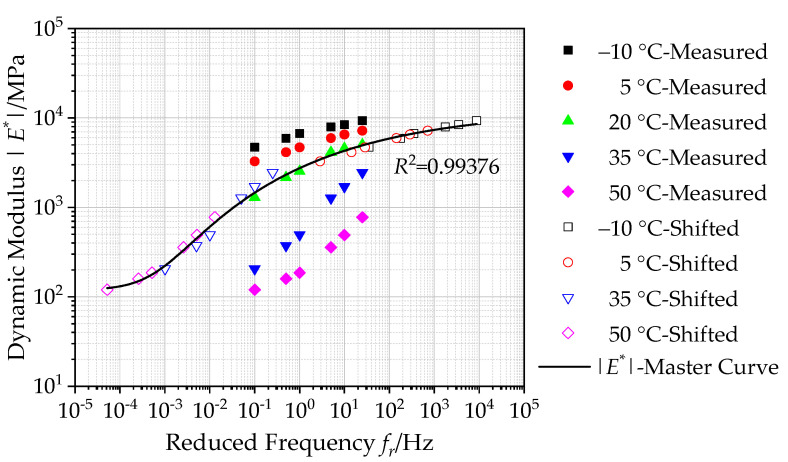
Master curve of dynamic modulus (reference temperature = 20 °C).

Figure 6 shows the calculated dynamic modulus |E*| together with the shifted test data and the constructed master curve of dynamic modulus. According to the calculated dynamic modulus |E*|, there is a significant change of dynamic modulus at a high frequency and high temperature. This could be attributed that the viscoelastic composition of asphalt materials changes at a high frequency and high temperature, and the more obvious the change, the higher the temperature and frequency. As the temperature increases or frequency decreases, the elastic behavior is weakened, and viscous behavior is more obvious [40].

Furthermore, based on Equation (10), the generalized Sigmoidal function is used to establish the master curve of dynamic modulus of asphalt mixtures incorporating of SBS polymer and basalt fiber at 20 °C in a log-log graph, as shown in Figure 6. It can be observed that the correlation coefficient *R*^2^ value of the generalized Sigmoidal model is determined to be 0.99376, which verifies that the calculated dynamic modulus |E*| in the master curve can match well with the shifted data. This also demonstrates the accuracy of the master curve of dynamic modulus. The calculated master curve of dynamic modulus can extend over a broader reduced frequency range at an arbitrary temperature taken as a reference, which is employed to reflect the dynamic modulus of asphalt materials incorporating of SBS polymer and basalt fiber accurately. In addition, the calculated master curve of dynamic modulus presented in Figure 6 shows an S-shaped growth trend, for which there is a changing trend of slow at both ends and fast at the middle overall, that is the variation of dynamic modulus slows down in a high-frequency and low-frequency range.

### 4.2. Determination of Phase Angle of Asphalt Mixture Reinforced with Basalt Fiber

#### 4.2.1. Influence Analysis of Temperature and Frequency on Phase Angle

During the cyclically dynamic loading, there is a stress-strain lag in asphalt mixture, in which this lag will change accordingly with the loading frequency. Similarly, as a typical viscoelastic material, this lag phenomenon will also change accordingly with the test temperature. This stress-strain lag could be characterized by phase angle.

**Figure 7:**
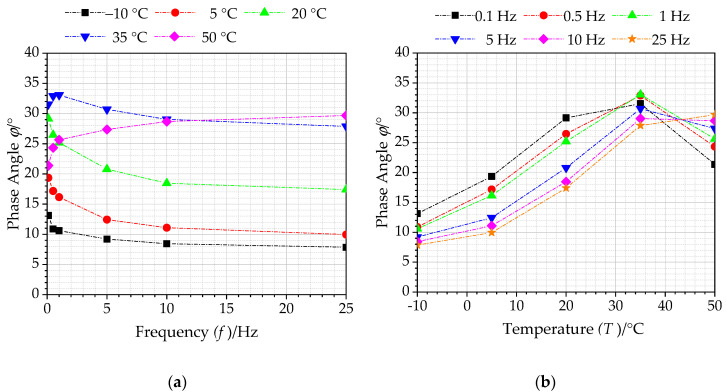
Phase angles of asphalt mixtures with basalt fiber: (**a**) temperature, (**b**) frequency.

Figure 7 presents the phase angle *ϕ* versus temperature and frequency for asphalt mixtures reinforced with basalt fiber. As observed in Figure 7a, the phase angle presents a different variation trend with loading frequency for different temperatures. At high temperature (35 °C and above), the phase angle exhibits an increasing trend with loading frequency. In the case of the experimental temperature below 35 °C, the phase angle will decrease with the increase of loading frequency. The main reason for this phenomenon is that asphalt mixture mainly exhibits elastic characteristics at low temperature, and there is almost no phase angle under load. On the other hand, asphalt binder gradually softens as the test temperature increases, resulting in a larger phase angle. This is because the phase angle inside asphalt mixture is attributed to the intrusion of aggregates, while the experimental temperature reaches 35 °C and above. Due to aggregate regarded as an elastic material, the phase angle will not be generated under load.

It can be observed from Figure 7b that phase angle varies with test temperature for different loading frequencies, but phase angle at each loading frequency would increase first and then decrease as the test temperature increases. For different loading frequencies, the corresponding temperatures at which the phase angle appears peak are different. This is because the bonding properties of asphalt bind is significantly affected by temperature, and the viscoelastic properties of asphalt mixtures are constantly changing with test temperature. At lower temperatures, the mechanical performances of asphalt materials are mainly determined by asphalt binder, while the impact of aggregate is relatively small. At this state, asphalt mixture is regarded as an elastic material and its phase angle is small. As the test temperature increases, asphalt mixture gradually tends to be viscous, and its phase angle increases accordingly. However, asphalt binder in asphalt mixture would soften as the test temperature continues to increase. Dynamic modulus mainly depends on aggregates inside asphalt mixture. Therefore, the phase angle will show a decreasing trend at higher temperature.

#### 4.2.2. Construction of Master Curves of Phase Angle

**Figure 8:**
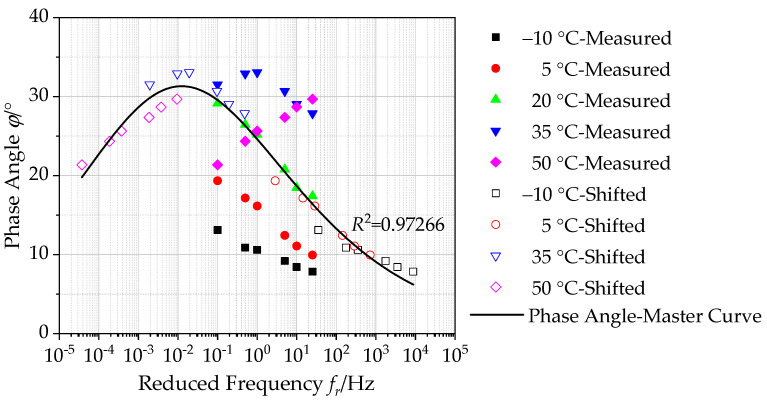
Master curve of phase angle (reference temperature = 20 °C).

Figure 8 plots the phase angle *ϕ* together with the shifted test data and the constructed master curve of phase angle. Based on the measured phase angle data, at various test temperatures or frequencies, the phase angle of asphalt mixture shows different variation trends. This could be attributed to the significant impact of temperature on asphalt binder in mixture. The viscoelastic behavior of asphalt mixture varies with test temperature, affecting its phase angle. At low temperature and high frequency, asphalt mixture mainly exhibits an elastic mechanical behavior, so the phase angle decreases with increasing frequency. However, at a high temperature and low frequency, asphalt binder softens and aggregate in asphalt mixtures dominates. Therefore, its phase angle gradually decreases with decreasing frequency.

The generalized Sigmoidal function in Equation (11) is employed to establish the master curve of phase angle of asphalt mixtures incorporating of SBS polymer and basalt fiber at 20 °C in a semi-log graph, as illustrated in Figure 8. It is observed that the correlation coefficient *R*^2^ value of the generalized Sigmoidal model is determined to be 0.97266, which verifies that the measured phase angle in the master curve can match well with the shifted data. This also demonstrates the accuracy of the master curve of phase angle. The calculated master curve of phase angle can extend over a broader reduced frequency range at an arbitrary temperature taken as a reference, which is employed to reflect the phase angle of asphalt mixtures incorporating of SBS polymer and basalt fiber accurately. In addition, the master curve of phase angle presented in Figure 8 shows a variation trend of increasing first and then decreasing.

### 4.3. Determination of Storage Modulus and Loss Modulus of Asphalt Mixture Reinforced with Basalt Fiber

On the basis of Equations (3) and (5), the storage modulus and loss modulus can be derived by using the calculated magnitude and phase angle in Equations (14) and (15) of complex modulus

(16) $$E^{\prime }=\left|E^{*}\right|\cos\varphi ,$$

(17) $$E^{\prime \prime }=\left|E^{*}\right|\sin\varphi ,$$

where *E*’ and *E*” are storage modulus and loss modulus, which characterize the pure elasticity and pure viscous mechanical behavior of asphalt mixtures, respectively.

#### 4.3.1. Storage Modulus and Master Curve of Asphalt Mixture with Basalt Fiber

**Figure 9:**
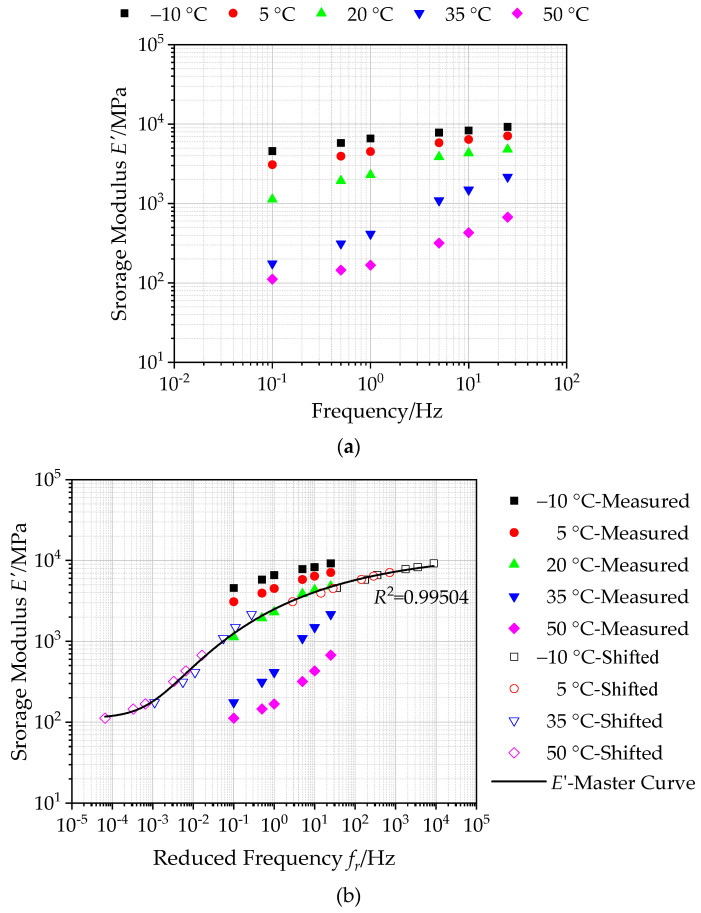
Storage modulus of asphalt mixtures with basalt fiber: (**a**) calculated storage modulus, (**b**) master curves of storage modulus (reference temperature = 20 °C).

Figure 9a illustrates the storage modulus *E’* versus temperature and frequency for asphalt mixtures reinforced with basalt fiber. As seen in Figure 9a, the storage modulus of asphalt mixtures exhibits a similar developing trend with dynamic modulus. At different loading frequencies, the storage modulus gradually decreases with increasing temperature, while the storage modulus increases with increasing loading frequency whatever the test temperature is. There is a significant change and its rate of storage modulus at a high frequency and high temperature. This could be attributed that the viscoelastic composition of asphalt mixtures changes at a high frequency and high temperature, and the elastic behavior is weakened.

Based on the calculated storage modulus, the generalized Sigmoidal model of storage modulus of asphalt mixtures incorporating of SBS polymer and basalt fiber was established at 20 °C, following Equation (12), which is plotted as a log-log graph in Figure 9b. It can be seen that the correlation coefficient *R*^2^ value of the generalized Sigmoidal model is determined to be 0.99504, which verifies that the calculated storage modulus in the master curve can match well with the shifted data. This also demonstrates the accuracy of the master curve of storage modulus. Simultaneously, the master curve of storage modulus in Figure 9b shows a similar S-shaped growth trend with the master curve of dynamic modulus. The calculated master curve of storage modulus can extend over a broader reduced frequency range at an arbitrary temperature taken as a reference, which is employed to reflect the storage modulus of asphalt mixtures incorporating of SBS polymer and basalt fiber accurately.

#### 4.3.2. Loss Modulus and Master Curve of Asphalt Mixture with Basalt Fiber

**Figure 10:**
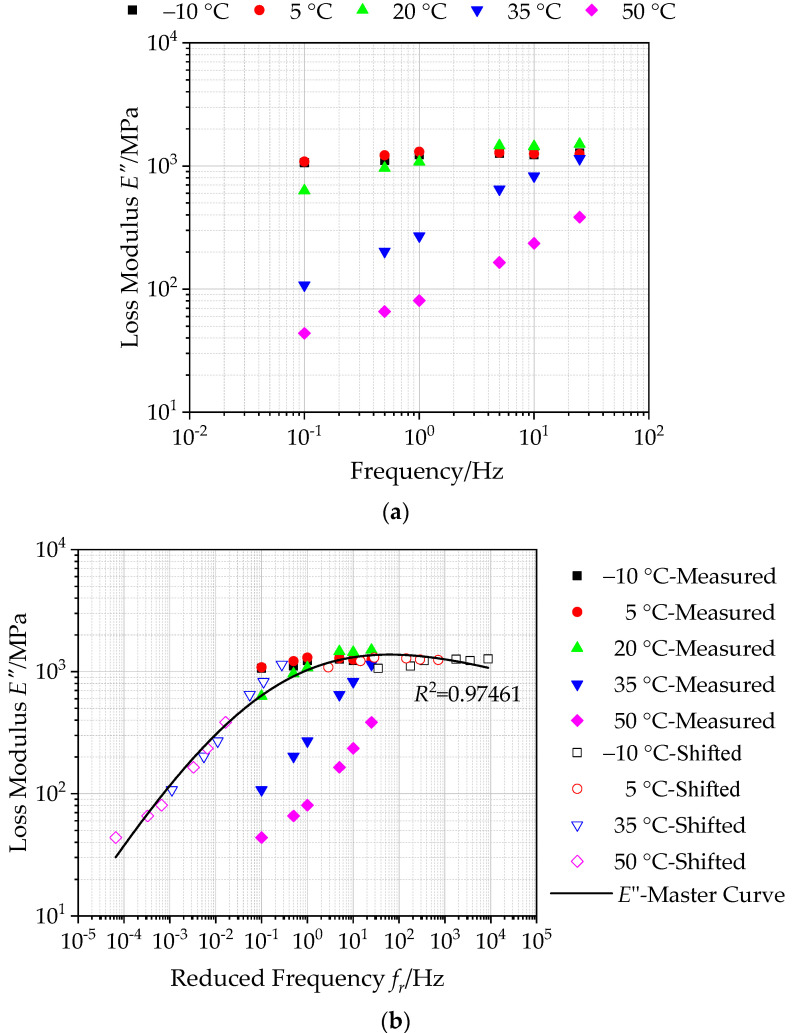
Loss modulus of asphalt mixtures with basalt fiber: (**a**) calculated loss modulus, (**b**) master curves of loss modulus (reference temperature = 20 °C).

Figure 10a illustrates the loss modulus *E”* versus temperature and frequency for asphalt mixtures incorporating of SBS polymer and basalt fiber. As illustrated in Figure 10a, the loss modulus of asphalt mixtures exhibits an increasing trend with the increasing of loading frequency at low temperatures. Whilem at high temperature, the loss modulus increases first and then decreases with increasing loading frequency.

Similarly, based on the calculated loss modulus, the generalized Sigmoidal model of loss modulus of asphalt mixtures incorporating of SBS polymer and basalt fiber was established at 20 °C based on Equation (13), which is plotted as a log-log graph in Figure 10b. It can be seen that the correlation coefficient *R*^2^ value of the generalized Sigmoidal model is determined to be 0.97461, which verifies that the calculated loss modulus in the master curve can match well with the shifted data. This also demonstrates the accuracy of the master curve of loss modulus. The calculated master curve of loss modulus can extend over a broader reduced frequency range at an arbitrary temperature taken as a reference, which is employed to reflect the loss modulus of asphalt mixture reinforced with basalt fiber accurately. In addition, the master curve of loss modulus in Figure 10b shows a variation trend of increasing first and then decreasing with the reduced frequency.

## 5. Conclusions

This paper aims to establish and evaluate the master curve of complex modulus of asphalt mixtures incorporating of SBS polymer and basalt fiber by using the generalized Sigmoidal model. Based on this research, the following conclusions can be drawn:

- Dynamic modulus values of asphalt mixture specimens exhibit an increasing trend with loading frequency. Under the same load, the dynamic deformation response would change with loading frequency, affecting the dynamic modulus.
- The dynamic modulus of asphalt mixture with basalt fiber decreases with the increasing of the test temperature. Asphalt mixture is closer to a linear elastomer at lower temperature, and it is prone to cracking and failure under a long-term load. While asphalt mixture would be prone to a large permanent deformation under the heavy load at higher temperature, due to the viscoelastic composition change. This could explain the rutting destruction for basalt fiber modified asphalt pavement.
- The phase angle varies with test temperature for different loading frequencies, but phase angle at each loading frequency would increase first and then decrease as test temperature increases. For different loading frequencies, the corresponding temperatures at which the phase angle appears peak are different.
- The storage modulus of asphalt mixture exhibits a similar variation trend with dynamic modulus. The storage modulus gradually decreases with increasing temperature, while the value increases with increasing loading frequency. With respect to loss modulus, it exhibits an increasing trend with the loading frequency at low temperatures. While, at high temperature, loss modulus increases first and then decreases. These moduli also show the variation trends of elastic and viscous behaviors of asphalt mixture.
- The generalized Sigmoidal model is proved to characterize the dynamic modulus, phase angle, storage modulus and loss modulus of asphalt mixture with basalt fiber with correlation coefficients above 0.97, which is utilized to predict the dynamic mechanical performances accurately. The established master curves can extend over a broader reduced frequency range at an arbitrary reference temperature accurately. The developed dynamic modulus model could provide a guidance for the repair and maintenance of basalt fiber reinforced asphalt pavement.

## Figures and Tables

**Table 1:** Technical parameters of styrene-butadiene-styrene (SBS) modified asphalt.

Test Parameters	Unit	Standards	Requirements	Values
Penetration	0.1 mm (@ 25 °C, 100 g, 5 s)	T0604	60~80	71
Ductility	cm (@ 15 °C, 5 cm/min)	T0605	≥30	45
Softening point	°C	T0606	≥55	60.5
Density	g/cm^3^	T0603	—	1.018
Flash point	°C	T0611	≥230	262
RTFOT				
Mass loss	%	T0609	±1.0	−0.094
Penetration ratio	% (@ 25 °C)	T0609	≥60	66.9

**Table 2:** Technical properties of basalt coarse aggregates.

Test Parameters		Unit	Standards	Requirements	Values
Crushing value		%	T0316	≤26	13.6
Los Angeles abrasion value		%	T0317	≤28	17.9
Apparent specific gravity	13.2 mm	—	T0304	≥2.6	2.836
	9.5 mm				2.805
	4.75 mm				2.726
Water absorption	13.2 mm	%	T0304	≤2.0	0.6
	9.5 mm				0.28
	4.75 mm				0.7
Soundness		%	T0314	≤12	5
Elongated particle content		%	T0312	≤15	9.2
Passing 0.075 mm sieve		%	T0310	≤1	0.3

**Table 3:** Technical properties of basalt fine aggregates.

Test Parameters	Unit	Standards	Requirements	Values
Apparent specific gravity	—	T0328	≥2.5	2.723
Water absorption	%	T0304	—	0.64
Angularity (flow time)	s	T0345	≥30	39.9
Sand equivalent	%	T0334	≥60	68

**Table 4:** Technical properties of limestone mineral filler.

Test Parameters		Unit	Standards	Requirements	Values
Apparent density		t/m^3^	T0352	≥2.5	2.712
Hydrophilic coefficient		—	T0353	<1	0.63
Water content		%	T0103	≤1	0.3
Plastic index		%	T0354	<4	2
Granular composition	<0.6 mm	%	T0351	100	100
	<0.15 mm			90~100	92.5
	<0.075 mm			75~100	81.8
